# *DRAXIN* as a Novel Diagnostic Marker to Predict the Poor Prognosis of Glioma Patients

**DOI:** 10.1007/s12031-022-02054-2

**Published:** 2022-08-30

**Authors:** Yulong Jia, Zhendong Liu, Xingbo Cheng, Runze Liu, Pengxu Li, Defu Kong, Wenjia Liang, Binfeng Liu, Hongbo Wang, Xingyao Bu, Yanzheng Gao

**Affiliations:** 1 Department of Neurosurgery, School of Clinical Medicine, Henan Provincial People’s Hospital, People’s Hospital of Zhengzhou University, Henan University, Zhengzhou, China; 2 Department of Orthopedics, School of Clinical Medicine, Henan Provincial People’s Hospital, People’s Hospital of Zhengzhou University, Henan University, Zhengzhou, Henan, China; 3 Department of Microbiome Laboratory, Henan Provincial People’s Hospital, People’s Hospital of Zhengzhou University, Zhengzhou University, Zhengzhou, Henan, China; 4 People’s Hospital of Zhengzhou University, Henan Provincial People’s Hospital, Zhengzhou, China; 5 People’s Hospital of Henan University, Henan Provincial People’s Hospital, Henan Province, 450003 China; 6 Department of Orthopedics, Henan Provincial People’s Hospital, Zhengzhou, Henan, China; 7 School of Basic Medical Sciences, Xinxiang Medical University, Xinxiang, 453003 China; 8 Department of Neurosurgery, Zhengzhou University People’s Hospital, Henan Provincial People’s Hospital, Zhengzhou, Henan, China; 9 Department of Surgery of Spine and Spinal Cord, Henan International Joint Laboratory of Intelligentized Orthopedics Innovation and Transformation, Henan Key Laboratory for Intelligent Precision Orthopedics, Henan Provincial People’s Hospital, People’s Hospital of Zhengzhou University, People’s Hospital of Henan University, Henan, Zhengzhou, 453003 China

**Keywords:** Gliomas, Biomarker, Oncogene, Cell cycle, Proliferation

## Abstract

An increasing number of evidences have shown that the carcinogenic effect of *DRAXIN* plays an important role in the malignant process of tumors, but the mechanism of its involvement in glioma has not yet been revealed. The main aim of this study is to explore the relationship between *DRAXIN* and the prognosis and pathogenesis of glioma through a large quality of data analysis. Firstly, thousands of tissue samples with clinical information were collected based on various public databases. Then, a series of bioinformatics analyses were performed to mine data from information of glioma samples extracted from several reputable databases to reveal the key role of *DRAXIN* in glioma development and progression, with the confirmation of basic experiments. Our results showed that high expression of the oncogene *DRAXIN* in tumor tissue and cells could be used as an independent risk factor for poor prognosis in glioma patients and was strongly associated with clinical risk features. The reverse transcription-quantitative PCR technique was then utilized to validate the *DRAXIN* expression results we obtained. In addition, co-expression analysis identified, respectively, top 10 genes that were closely associated with *DRAXIN* positively or negatively. Finally, in vitro experiments demonstrated that knockdown of *DRAXIN* significantly inhibited proliferation and invasion of glioma cell. To sum up, this is the first report of *DRAXIN* being highly expressed in gliomas and leading to poor prognosis of glioma patients. *DRAXIN* may not only benefit to explore the pathogenesis of gliomas, but also serve as a novel biological target for the treatment of glioma.

## Introduction

Gliomas are malignant tumor of neuroepithelial origin due to the interaction of congenital genetic risk factors and environmental carcinogenic factors, which frequently occur in brain and spinal cord glial cells. The annual incidence rate of glioma is 5.26 per 100,000 or 17,000 new cases diagnosed each year (Omuro and DeAngelis 2013). The diagnostic criteria of glioma based on pathological classification have gradually shifted from microscopic tumor morphology to a combination of morphological and molecular features, including gene mutations, chromosome copies, and gene rearrangements, etc. (Wood et al. 2019). The following standard treatments include surgical resection, chemotherapy, immunotherapy, and a number of innovative treatments known as tumor treatment field or photodynamic therapy (Agostinis et al. 2011; Liu et al. 2019). Although the above intervention methods for glioma have reached a comparatively high level, therapeutic effects still do not meet our expectations, probably due to chemoradiotherapy resistance or poor marker specificity. Consequently, there is an urgent need to find better biomarkers that can benefit the accurateness of diagnose and predict the prognosis of glioma.

To date, a number of biomarkers have been identified for the diagnosis and prognosis of glioma. Epidermal growth factor receptor (EGFR) is involved in tumorigenesis and multiple signaling pathways. Disregulated expression of EGFR occurs in approximately 40% of glioblastoma patients, and its mutation, EGFRvIII, reaches 20 to 30% of glioblastoma (Riemenschneider et al. 2010). Therefore, EGFR has a guiding role in the diagnosis of glioma, and EGFRvIII can be used to distinguish high-grade gliomas and poor prognosis of patients. Another valid biomarker CD133 is a membrane-bound protein encoded by the gene *PROM1*, which may play a key role in cell differentiation. It is reported that CD133-positive cells derived from human glioma cells are highly susceptible to replicate the original tumor, so increased proportion of CD133 cells indicates a lower survival rate. Thus, the mRNA level of encoding gene PROM1 can be used to differentiate between GBM and low-grade gliomas. There are also several well-known biomarkers such as CD15, A2B5, and Nestin that can benefit to increase the diagnosis and prognosis of glioma (Ludwig and Kornblum 2017). As the fact that many biomarkers have been identified, malignancies are inherited diseases caused by mutations in multiple genes. Therefore, one single gene is not enough to accurately predict and treat malignant tumor. In this study, we are committed to discovering novel predictors for glioma.

Dorsal rejection axon guidance protein (DRAXIN), encoded by the *DRAXIN* gene, is required for the development of spinal cord and forebrain connectivity, as a chemical driver-guiding protein for conjoined axons (Zhang et al. 2010; Sato et al. 2018). It can inhibit or repel the growth of dorsal spinal cord neurons. In recent years, the role of *DRAXIN* in tumors has gradually become apparent, as reported in lung cancer (Sato et al. 2018). However, the expression of *DRAXIN* in glioma has not been reported, so the present study is the first of its kind to clarify the relationship between *DRAXIN* and characteristics of glioma.

Firstly, we demonstrated that *DRAXIN* expression is much higher in glioma tissues and cell lines compared to normal samples. KM and ROC curve–exhibited *DRAXIN* was correlated with shorter OS, and univariate and multivariate regression analysis claimed that *DRAXIN* was an independent prognostic factor for glioma. We performed Pearson correlation coefficient to prove the positive relationship of *DRAXIN* and malignant clinical features. Furthermore, GSEA revealed potential signaling pathways that *DRAXIN* might participate in. At last, individually ten most-related genes and four candidate drugs for glioma were identified. It is believed that this study will provide *DRAXIN* as an effective biological target for the prognosis of glioma and may provide new research directions for the drug treatment of glioma.

## Materials and Methods

### Data Collection

GEPIA (Gene Expression Profiling Interactive Analysis) (http://gepia.cancer-pku.cn/) is a database established by Peking University that contains massive RNA sequencing data from human tumor tissues and mutually matched normal tissues, creating new opportunities for data mining and a deeper understanding of gene function (Tang et al. 2017). GEO (Gene Expression Omnibus) (https://www.ncbi.nlm.nih.gov/geo/) is an international public repository containing high-throughput microarray and next-generation sequence functional genomic datasets, containing data from multiple pathological and normal samples (Barrett et al. 2013). We screened and obtained the key dataset GSE50161, which contains genetic experimental microarray data from tumor tissues (n = 34) and normal control tissues (n = 13). The IVY-GAP (Ivy-Glioblastoma Atlas Project) (http://glioblastoma.alleninstitute.org/static/home) database is dedicated to GBM-related studies. Here, we obtained the characteristic annotation of *DRAXIN* in glioma tissues by ISH/FISH/HE for its expression level in gliomas.

CGGA (Chinese Glioma Genome Atlas) (http://www.cgga.org.cn/) is a database of gene sequencing and clinical characteristics of a large number of glioma patient samples established by Beijing Tiantan Hospital, Capital Medical University (Yan et al. 2012). A total of 748 samples were selected and analyzed by screening the glioma samples for complete clinical information (Table 1). Survival analysis, univariate Cox analysis, multivariate Cox analysis, co-expression analysis, and ROC curve analysis were performed on this basis.

**Table 1:** Characteristics of patients with glioma based on CGGA

**Characteristics**		**Number of cases**	**Percentages (%)**
Gender	Male	306	40.91
	Female	442	59.09
Age	< = 41	341	45.59
	> 41	407	54.41
Grade	WHO II	218	29.14
	WHO III	240	32.09
	WHO IV	290	38.77
PRS type	Primary	501	66.98
	Recurrent	222	29.68
	Secondary	25	3.34
Radio status	Yes	625	83.56
	No	124	16.58
Chemo status	Yes	520	69.52
	No	228	30.48
Histology	astrocytoma	55	7.35
	Anaplastic astrocytoma	39	5.21
	Anaplastic Oligodendroglioma	22	2.94
	Anaplastic oligoastrocytoma	80	10.70
	Glioblastoma	175	23.40
	Oligodendroglioma	35	4.68
	oligoastrocytoma	95	12.70
	relapse astrocytoma	20	2.67
	relapse Anaplastic astrocytoma	36	4.81
	relapse Anaplastic Oligodendroglioma	15	2.01
	relapse Anaplastic oligoastrocytoma	48	6.42
	relapse Oligodendroglioma	90	12.03
	relapse Oligodendroglioma	4	0.53
	relapse oligoastrocytoma	9	1.20
	Secondary relapse Oligodendroglioma	25	3.34
IDH mutation status	Mutant	409	54.68
	Mutant with 1p19q codeletion	146	19.52
	Mutant without 1p19q codeletion	263	35.16
	Wildtype	339	45.32
	Wildtype with 1p19q codeletion	9	1.20
	Wildtype without 1p19q codeletion	330	44.12
1p19q_codeletion_status	Non-codeletion	593	79.28
	Mutant without 1p19q codeletion	263	35.16
	Wildtype without 1p19q codeletion	330	44.12
	Codeletion	155	20.72
	Mutant with 1p19q codeletion	146	19.52
	Wildtype with 1p19q codeletion	9	1.20

### GSEA Analysis of DRAXIN

GSEA (Gene Set Enrichment Analysis) can be used for data analysis of whole genome expression profiles. It is used to compare genes with predefined gene sets (Subramanian et al. 2007). GSEA analysis was performed on mRNA sequencing data obtained from the CGGA database. First, batch calibration and normalization were performed by LIMMA packages. Then, the RNA sequencing data were divided into high expression group (H group) and low expression group (L group) according to the median value of *DRAXIN* expression. GSEA (version 4.0.3) jar software was used for enrichment analysis. The number of enrichment was set to 1000 times, and the KEGG cell signaling pathway was selected as the gene database. Normal *P* < 0.05 and FDR < 0.25 were regarded as significantly enriched.

### Tissue Collection

Clinical specimens of 17 glioma tissue samples and 7 normal brain tissues (Taken from normal brain tissue suitable for resection in patients with epilepsy) were obtained from patients in the clinical department of Henan Provincial People’s Hospital, and patients signed a written informed consent. Collected samples were stored in liquid nitrogen environment, and then transferred to − 80 °C freezer for use. This study was approved by the Ethics Committee of Henan Provincial People’s Hospital.

### Cell Culture and Treatment

Human glioma cell lines (U251, T98, A172) and human astrocytes (HA) were purchased from Shanghai Shunran HAKATA Cell Bank (http://www.xrshbio.com/). All cells were cultured in an incubator at 37 °C and 5% CO_2_ using DMEM medium (HyClone, USA) containing 10% fetal bovine serum (FBS, Gibco, USA). U251 cells with a more stable phenotype were selected for the in vitro experiments. U251 cells were cultured in 6-well plates and when the cells were fused to 70–80%, the cells were intervened with siRNA purchased by Genepharma. The siRNA specifically targeting *DRAXIN* (siDRAXIN) was Sense 5′-GCCCUCUGCAAAGAAGAAATT-3′, Antisense 5′-UUUCUUCUUUGCAGAGGGCTT-3′. The siRNA used as negative control (siNC) was Sense 5′-UUCUCCGAACGUGUCACGUTT-3′, and Antisense5′-ACGUGACACGUUCGGAGAATT-3′. After incubated for 48 h, U251 cells were applied to further experiments.

### RNA Extraction and Quantitative Reverse Transcription Polymerase Chain Reaction (RT-qPCR) Analysis

RT-qPCR was used to detect the expression of *DRAXIN* in glioma tissues and glioma cell lines. Tissue RNA and cellular RNA were extracted with Tri-Reagent and QIA symphony™ RNA kit (QIAGEN, Germany). Subsequently, the RNA solubility was determined using an ultra-micro spectrophotometer (Thermo Fisher, USA). The NovoStart SYBR qPCR SuperMix Plus (Novoprotein, China) kit was used to perform RT-qPCR. GADPH was used as an internal reference. The primer sequences used for *DRAXIN* were 5′-CGACTGGACCGATTATGAAGAC-3′ (F) and 5′-CGGCTGGTGATGTTTCGTTAC-3′ (R). “2^−ΔΔCT^” or “2^−ΔCT^” methods were individually applied to analyze the *DRAXIN* expression of glioma cells and tissues. The unpaired *t* test was used for the statistical difference between the two groups. When the *p* value was < 0.05, the difference between the two groups was statistically significant.

### CCK8 Assay and Colony Formation Assay

U251 cells treated with siRNA were cultured in the 96-well plate with a density of 2000 cells per well. At the set time point, the working solution of Cell Counting Kit-8 was added into the 96 well plate. After continuous incubation at 37 ℃ for 4 h, the absorbance value of U251 cells was detected.

Transfected cells were cultured at a density of 500 cells per well in 6-well plates and continued to be cultured under complete medium conditions for 14 days. The next step, colony-forming cells were washed with PBS and fixed using paraformaldehyde for 30 min. Crystal violet solution was used to stain cell colonies, and finally the number of colonies was counted and statistically analyzed.

### Transwell Assay and Wound Healing Assay

Cells were treated with siRNA and cultured in transwell chambers at a density of 1000 per well in the culture medium with 5% FBS. Then, the medium with 20% FBS was added in the lower chamber of the 24-well plate. After 48 h of incubation, cells invading the lower sidewalls of the transwell chambers were fixed and stained. The number of invading cells was recorded and used for statistical analysis.

### Immunohistochemistry

Normal brain tissues and glioma tissues with different grades from laboratory were used for immunohistochemical staining. First, samples were dewaxed using xylene and dehydrated using gradient ethanol. After antigen repair and endogenous peroxidase blockade, the samples were sealed by 10% BSA solution. Next, specific antibody targeting *DRAXIN* (1:500, Bioss, China) was used to incubate the samples overnight at 4 °C. Further incubation with secondary antibody, samples were dyed via Mouse/Rabbit enhanced polymer detection system (ZSGB-BIO, China), and then nuclei in samples were stained with hematoxylin. Finally, the protein expression of *DRAXIN* was photographed by a microscope and analyzed by ImagePro-Plus software (version 6.0).

### Statistical Analysis

The R software (v.4.0.3) was frequently used to analyze the above multiple sets of data and perform statistical analysis. The Wilcox test was used to determine the expression of *DRAXIN* in glioma and non-tumor brain tissues. Cox regression and Kaplan–Meier method were used to examine the relationship between the expression level of *DRAXIN* and the patient’s OS, and to draw survival curves and ROC curve. Univariate Cox analysis and multivariate Cox analysis based on statistical data sheet was performed. Use Wilcox or Kruskal test to detect the relationship between clinically relevant information and *DRAXIN* in patients with glioma expression, *p* < 0.05 is statistically significant.

## Results

### Clinical Characteristics Information

In total, the 748 cases of glioma transcriptomic data from the CGGA database were collected in the present study. These data include general clinical information of glioma patients, such as gender, age, pathological type, and WHO classification of glioma, in addition to PRS (primary, recurring, secondary) type, IDH (isocitrate dehydrogenase) mutation and some data on the missing status of the 1p19q co-deletion. Detailed clinical information is shown in Table 1.

### The Expression of DRAXIN is Higher in Glioma Tissues than in Normal Control

**Fig. 1:**
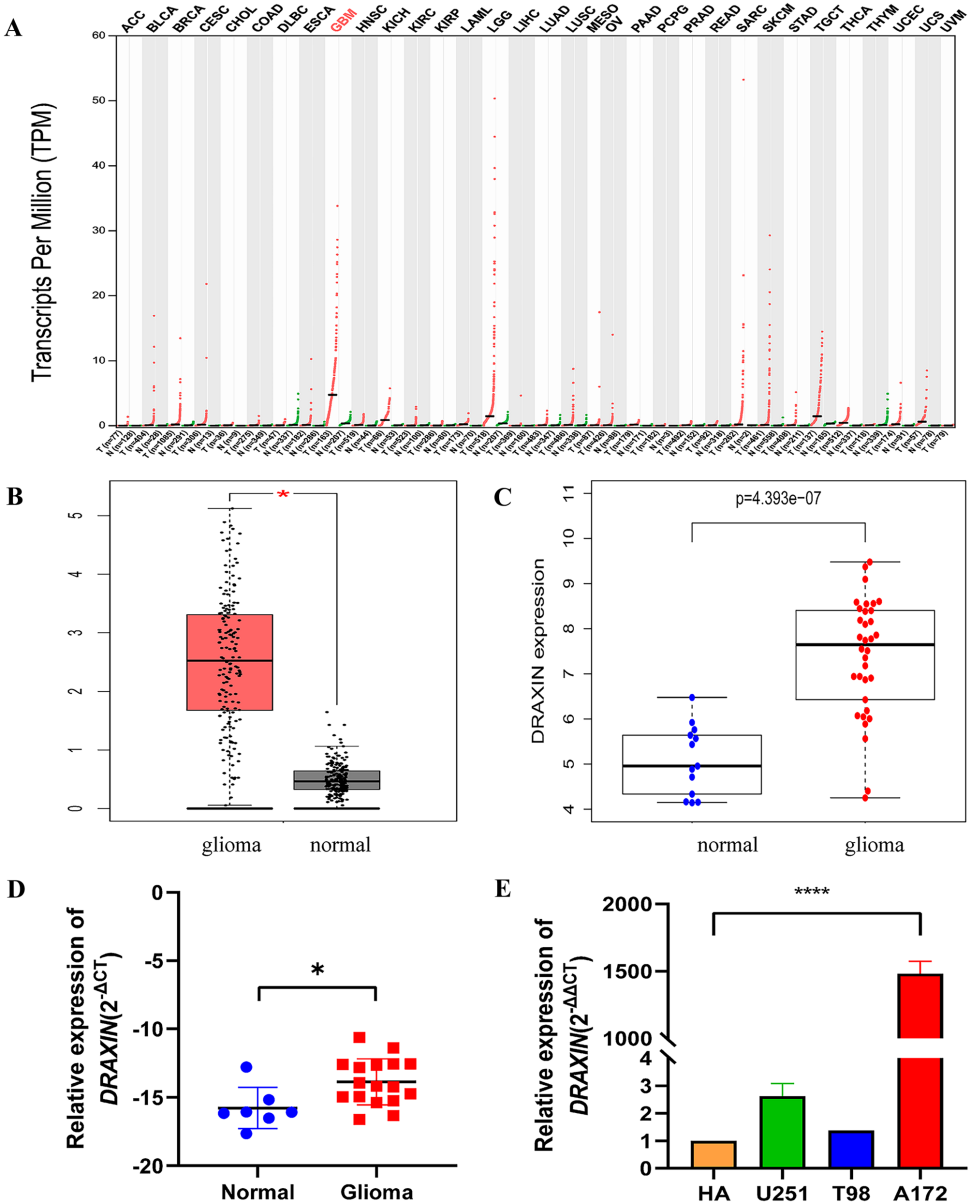
Comparison of *DRAXIN* expression levels in glioma and normal tissues at different levels. **A** Based on the GEPIA database, differences in the expression levels of *DRAXIN* in various tumors. *DRAXIN* is highly expressed in GBM (n = 163) compared to normal tissues (n = 207). The different colors of the tumor name represent different meanings. Red means that the expression of *DRAXIN* in tumor tissue is higher than that of normal tissue, Black indicates that the expression of *DRAXIN* in tumors is not statistically significant compared to the expression of normal tissues; **B** Based on the GEPIA database, the expression level of *DRAXIN* gene in GBM (n = 163) and corresponding normal tissues (n = 207) expression level box plot; **C** The expression level of *DRAXIN* gene in glioma (n = 34) and corresponding normal tissues (n = 13) based on GSE50161; **D** Based on RT-qPCR, the expression level of *DRAXIN* in glioma and corresponding normal tissues; **E** The expression level of *DRAXIN* gene in the glioma cell lines U251, T98, A172 and the corresponding normal control cell line (HA) based on RT-qPCR (*p* < 0.05)

**Fig. 2:**
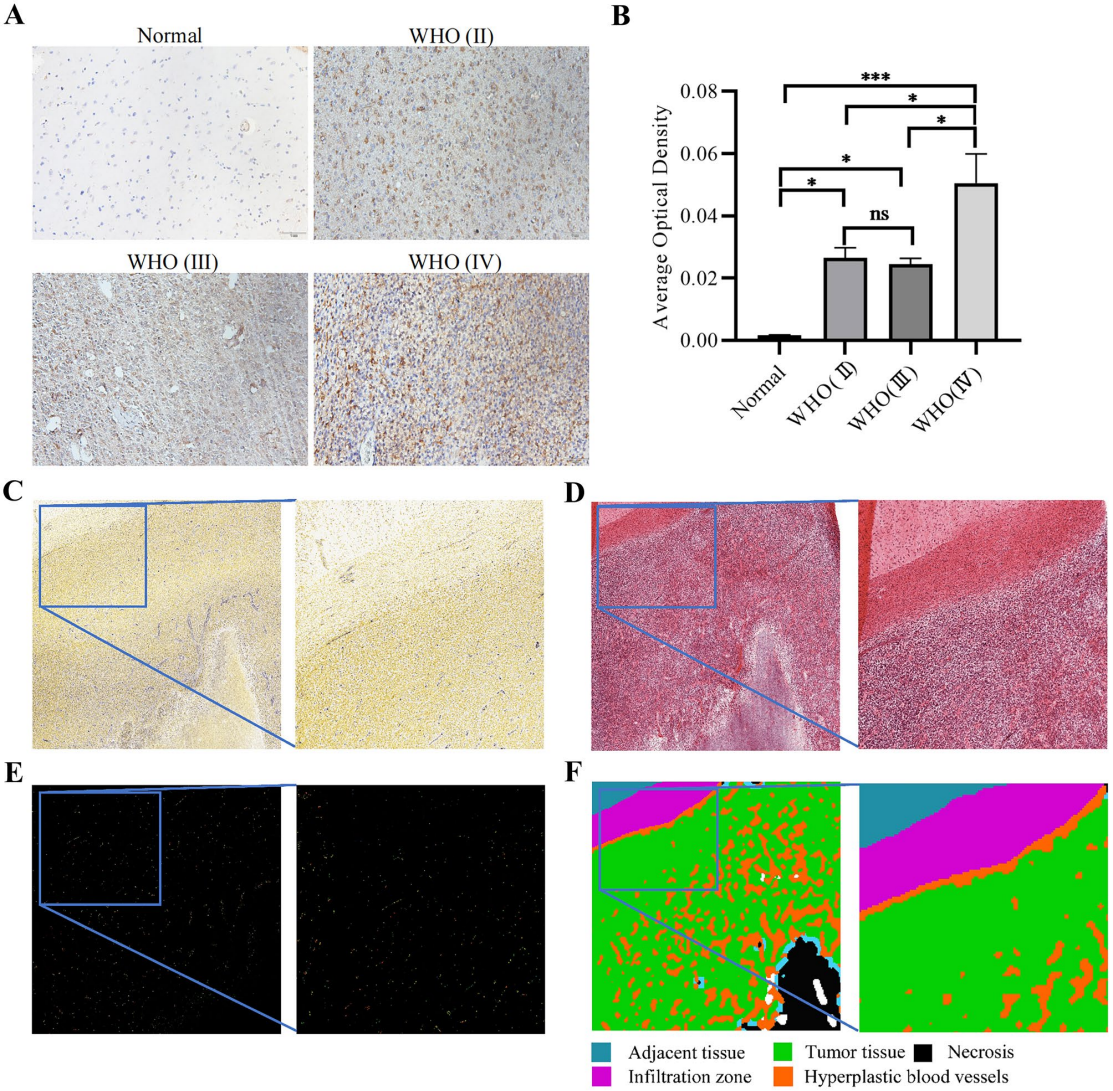
High expression of *DRAXIN* based on glioma tissues from laboratory and the IVY-GAP database. **A**–**B** IHC results show the expression of *DRAXIN* in normal brain tissue and glioma tissues with different WHO grades. **C**–**F** The expression of *DRAXIN* shown by in situ hybridization, **H**&**E** staining technique; fluorescence in situ hybridization; and tumor feature annotation. Green represents tumor tissue. Blue represents advanced tissue. Purple represents the infiltration zone. Black represents necrosis. Orange represents hyperplastic blood vessels

The study first explored the expression levels of *DRAXIN* in a variety of tumors using the GEPIA database, and the results showed that *DRAXIN* was significantly highly expressed in GBM (Fig. 1A). In addition to statistical differences in the GEPIA database (Fig. 1B), the study showed consistent results in the GSE50161 dataset (Fig. 1C). Second, the experiments further verified that mRNA levels of *DRAXIN* were increased in gliomas using laboratory brain tissue samples (Fig. 1D), and were also significantly higher in glioma cell lines, especially in A172 cells, compared to HA cell (Fig. 1E). At the tissue level, IHC results demonstrated that protein levels of *DRAXIN* increased in glioma samples in comparison with normal brain tissue and was the highest in Grade IV glioma (Fig. 2A–B). Furthermore, the high expression of *DRAXIN* in GBM was exhibited by the IVY-GAP database using ISH images (Fig. 2C–F). Overall, the experiments confirmed the high expression of *DRAXIN* in glioma through multidimensional evidence.

### Relationship Between the Expression of DRAXIN and Prognosis of Patients with Glioma

**Fig. 3:**
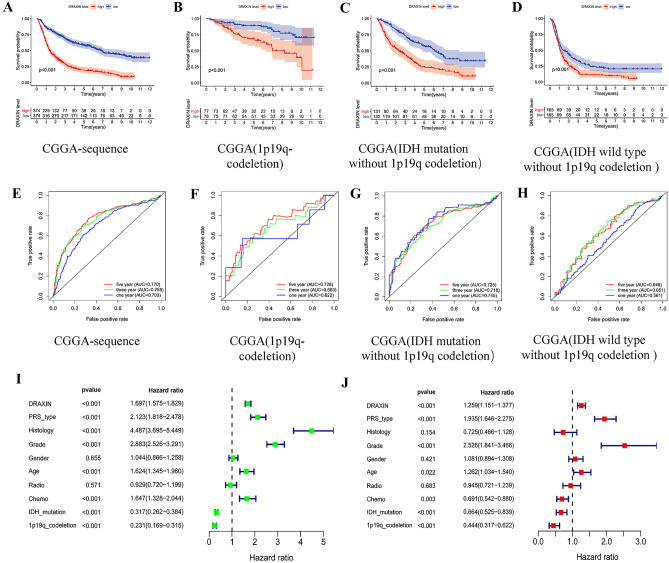
Based on the CGGA database, the high expression of *DRAXIN* gene can be used as an independent risk factor to cause poor prognosis, and can be used as a prognostic assessment method; **A**–**D** Kaplan–Meier survival curve based on CGGA database shows that patients with four different molecular subtypes with high expression of *DRAXIN* have a lower survival rate; **E**–**H** ROC curve shows that the expression of *DRAXIN* has a higher prognosis for the 5-year survival rate of glioma patients assessment value (AUC > 0.7); **I** univariate cox analysis of the prognosis of glioma patients; **J** multivariate cox analysis of the prognosis of glioma patients

To further explore whether the high expression of *DRAXIN* has a certain clinical significance, such as a certain impact on the prognosis, OS curve was drawn and it was found that the survival time of the high expression group was lower than the low expression group in these queues, indicating a poor prognosis (Fig. 3A). In addition, combined with the conditions such as WHO classification, IDH mutation, and 1p19q co-deletion status, OS diagrams were further obtained (Fig. 3B–D). Moreover, ROC curves were drawn with above categories, we discovered that expression of *DRAXIN,* accompanied with 1p19q no-deletion status, and IDH mutation with 1p19q co-deletion status has statistical significance (AUC > 0.7). In the end, univariate and multivariate logistic regression analysis exhibited three independent risk factors (HR > 1, *p* < 0.001) for glioma, the expression of *DRAXIN*, PRS grade and high-grade glioma leading to poor prognosis (Fig. 3I, J). IDH mutation type and 1p19q co-deletion status were present as protective factors (HR < 1, *p* < 0.001). Based on the above data, high expression of *DRAXIN* could be used to indicate a poor prognosis for glioma patients, and it had a certain clinical diagnostic value.

### The Correlation Between the Expression of DRAXIN and Clinical Features in Patients With Glioma

**Fig. 4:**
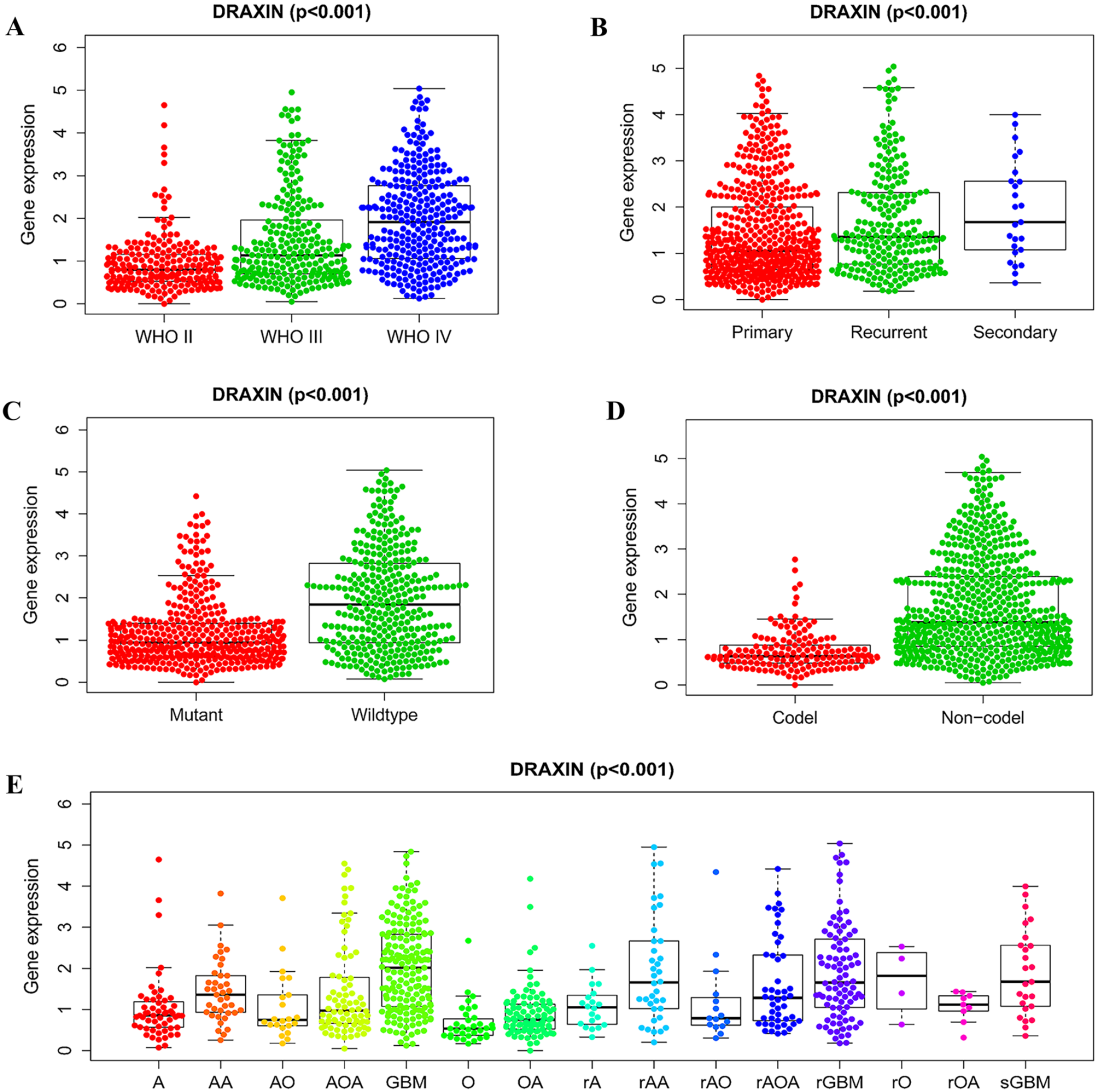
The expression of *DRAXIN* in glioma is related to a variety of clinical features. **A** WHO classification; **B** PRS classification; **C** IDH mutation status; **D** 1p19q co-deletion status; **E** pathological tissue typing

Not only of overall survival, the relationship between various clinical characteristics and expression of *DRAXIN* was also estimated via correlation analysis. The data of glioma was screened for the complete information including gender, age, pathological type, and WHO grade of glioma, PRS (primary, recurring, secondary) type, IDH (isocitrate dehydrogenase) mutation and the status of the 1p19q codon. After Wilcox and Kruskal tests, it was concluded that the increased expression of *DRAXIN* was positively correlated with higher WHO grade and recurrent and secondary glioma (*p* < 0.001; Fig. 4A–B). In the primary glioma, the expression of *DRAXIN* in IDH wild-type was higher than that of the mutant (*p* < 0.001, Fig. 4C). Compared to gliomas with 1p19q co-deletion status, the expression level of *DRAXIN* was higher in that with non-co-deletion state (*p* < 0.001, Fig. 4D). In addition, histocytology results showed that the expression level of *DRAXIN* in the GBM group and the relapsed GBM group was significantly increased. In summary, the expression of *DRAXIN* was significantly related to the malignant features of glioma.

### GSEA Analysis and Co-expression Analysis of DRAXIN

**Fig. 5:**
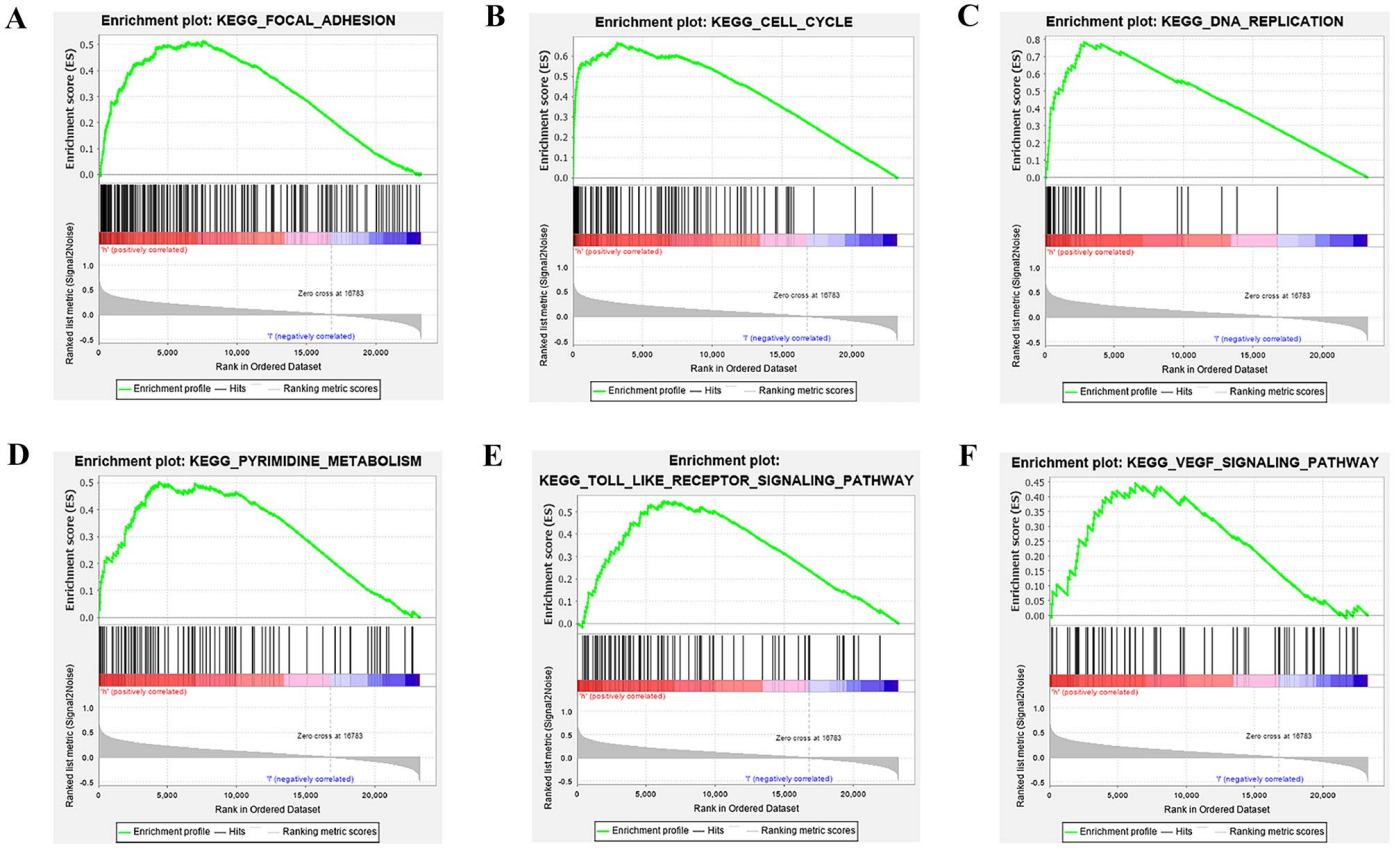
GSEA enrichment analysis results of *DRAXIN*. **A** Focal adhesion, **B** Cell cycle, **C** DNA replication, **D** Pyrimidine metabolism, **E** Toll-like receptor signaling pathway, **F** VEGF signaling pathway

In order to achieve the purpose of revealing the specific mechanism of *DRAXIN* in glioma, GSEA analysis was firstly applied to reveal the cancer-related cell signaling pathways that *DRAXIN* might involve in. Analysis results were obtained by screening with *p* < 0.05, FDR *q*-value < 0.25, which turned out that focal adhesion, cell cycle, DNA replication, pyrimidine metabolism, Toll-like receptor signaling pathway, VEGF signaling pathway, in which the group with high expression of *DRAXIN* showed significant enrichment (Fig. 5). The above results suggest that *DRAXIN* may be involved in the malignant progression of glioma through the mentioned signaling pathways.

**Fig. 6:**
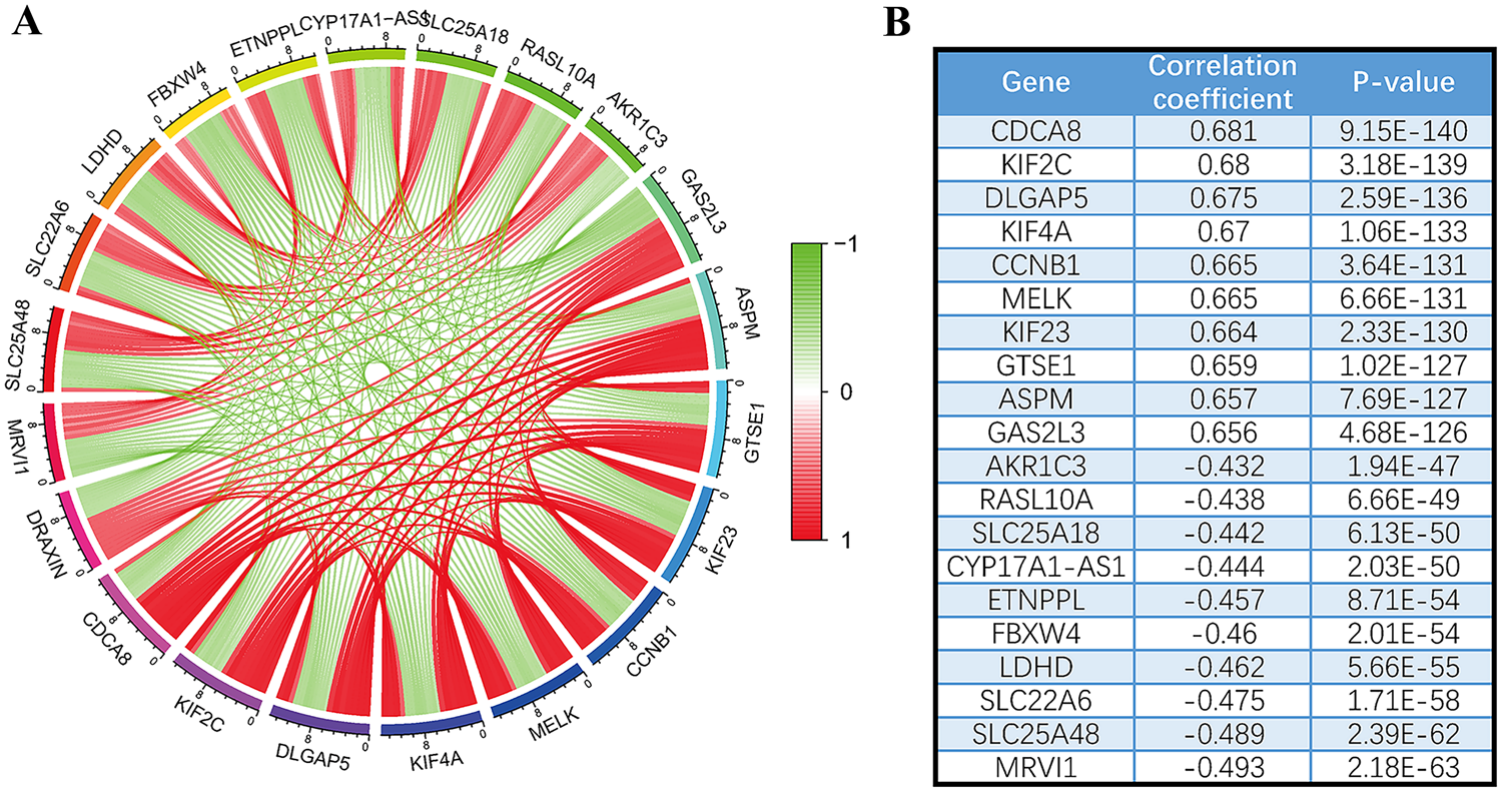
The co-expression analysis results of *DRAXIN* gene and 20 related genes in glioma. **A** Twenty genes are positively correlated and negatively correlated with the expression of *DRAXIN*. **B** 20 related genes of *DRAXIN* and their correlation coefficients

Furthermore, to explain the role of *DRAXIN* itself in glioma, individual top ten most related genes were obtained after screening the *p* value and correlation coefficient value of co-expressed genes (Fig. 6). Ten genes were positively correlated with *DRAXIN*, including *CDCA8*, *KIF2C*, *DLGAP5*, *KIFA4*, *CCNB1*, *MELK*, *KIF23*, *GTSE1*, *ASPM*, and *GAS2L3*, and other ten genes, *AKR1C3*, *RASL10A*, *SLC25A18*, *CYP17A1-AS1*, *ETNPPL*, *FBXW4*, *LDHD*, *SLC22A6*, *SLC25A48* and *MRVI1*, were negatively correlated with *DRAXIN*. Results of co-expressed genes suggest potential molecular functions of *DRAXIN* in glioma.

### Knockdown of DRAXIN Expression Inhibits the Proliferative and Invasive Capacity of Glioma Cells

**Fig. 7:**
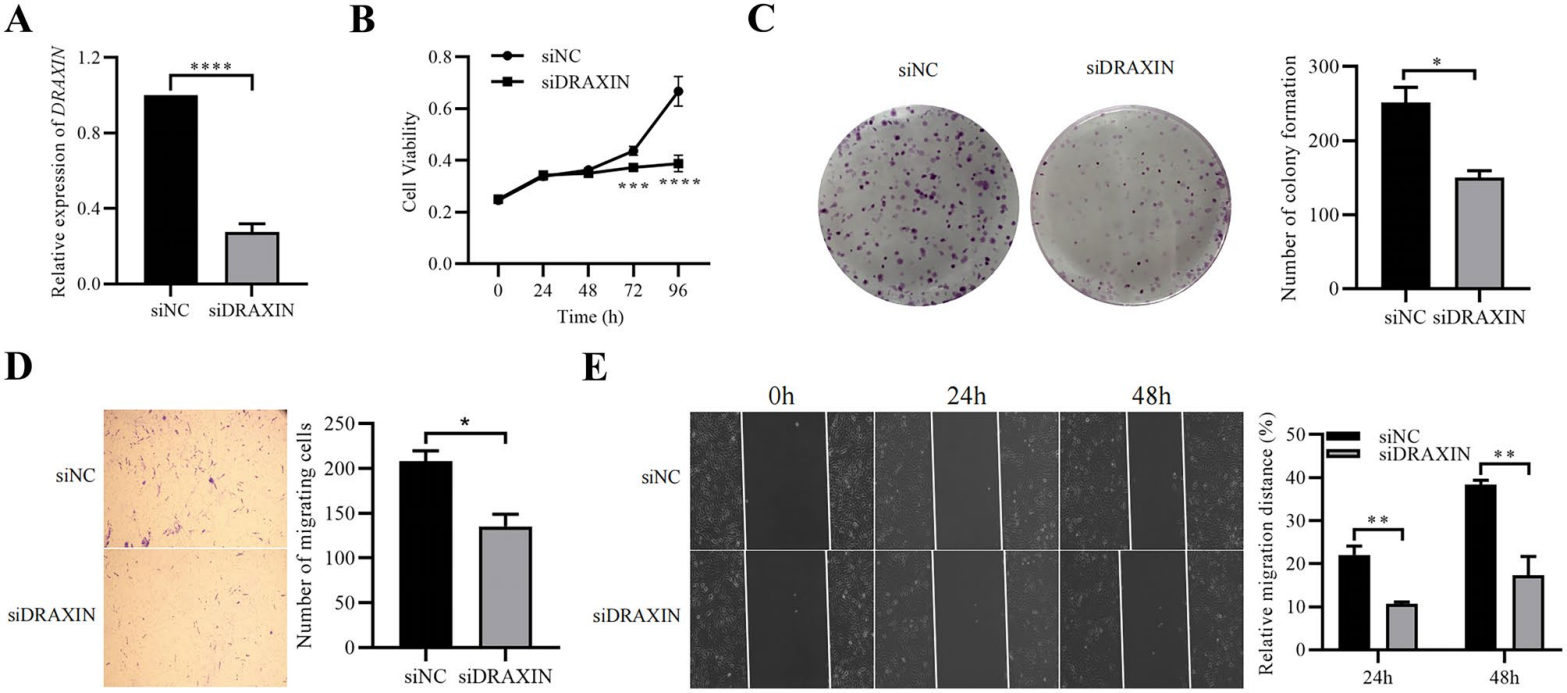
Knockdown of *DRAXIN* remarkably inhibits proliferation and invasion of U251 cells. **A** The mRNA expression of *DRAXIN* in siNC group and siDRAXIN group. **B** The cell viability of both siRNA-treated groups at different time points. **C** Staining results of colony formation of siRNA-treated groups. **D** Staining of invading cells of both groups in the transwell assay. **E** Relative distances of both siRNA treated groups in wound healing assay. * *p* < 0.05, ** *p* < 0.01, *** *p* < 0.001, **** *p* < 0.0001

To verify the role of *DRAXIN* in glioma and further increase the scientific rigor of the study, in vitro experiments first knocked down the expression of *DRAXIN* in U251 cells. As shown in Fig. 7A, the mRNA level of *DRAXIN* was reduced by nearly 70% in siDRAXIN group compared to siNC group. CCK8 experiments confirmed that the proliferation ability of cells in siDRAXIN group was significantly decreased at 72 h (*p* < 0.001) and 96 h (*p* < 0.0001) (Fig. 7B). Also, the number of cell colonies in siDRAXIN group was also significantly lower than that in siNC group (*p* < 0.05) (Fig, 7C). Both experiments indicated that knockdown of *DRAXIN* significantly inhibited the proliferation ability of glioma cells. Furthermore, the number of invading cells in the transwell assay (*p* < 0.05) and the relative cell migration distance in the wound healing assay (*p* < 0.01) were both significantly lower in the siDRAXIN group than in the siNC group (Fig. 7D–E). Overall, *DRAXIN* does promote cell proliferation capacity and invasive ability in glioma.

## Discussion

*DRAXIN* is commonly recognized as an axon guidance cue for thalamocortical development (Shinmyo et al. 2015). It can regulate hippocampal neurogenesis through the inhibition of neuroblasts apoptosis (Tawarayama et al. 2018). When this idea is extended to tumor field, *DRAXIN* is reported to be a key factor in the progress of lung adenosquamous carcinoma via the regulation of cell proliferation and apoptosis (Sato et al. 2018). Nevertheless, the molecular mechanism of *DRAXIN* in glioma has not been reported. The aim of this study was to reveal the function of *DRAXIN* in glioma from multiple perspectives and its impact on patient treatment and prognosis.

The rigor of the analysis for aberrant expression of *DRAXIN* in glioma was improved by validation using GEPIA, CGGA database, IVY-GAP database and basic experimental RT-qPCR and IHC. High expression of oncogenes is often closely associated with poor prognosis and malignant features of tumors (Li et al. 2018). Our study also demonstrated the consistent results by plotting survival curves, univariate and multivariate Cox analysis, as well as correlation coefficient analysis. Besides the correlation with shorter OS, *DRAXIN* expression was found positively correlated with glioma WHO Grade, 1p19q non-co-deficiency, GBM and recurrent GBM. Interestingly, these factors are reported to act as independent risk factors for glioma. (Ducray et al. 2008; Louis et al. 2016). For example, in 1p/19q non-codel gliomas, the expression of 1p19q is highly associated with the poor prognosis and malignancy of the tumor (Chai et al. 2019). After we attempted to group the overall samples according to two factors, 1p19q and IDH, subsequent survival analysis and ROC curves showed that abnormally high *DRAXIN* expression were still significantly associated with poor prognosis. This result strongly suggests that *DRAXIN* is a novel prognostic factor for glioma beyond 1p19q co-deletion and IDH mutant.

To further discover the potential mechanism, GSEA analysis was performed to reveal the possible signaling pathways of *DRAXIN* that might participate in leading to the carcinogenesis of glioma. Enrichment results showed cell cycle, DNA replication, pyrimidine metabolism, VEGF signaling pathway, focal adhesion, and Toll-like receptor signaling pathway, which had influential functions in the development of glioma. First of all, the cell cycle signaling pathway exists throughout the cell division cycle and is activated in almost all tumors (Liu et al. 2019). Aberrant anabolism of DNA and proteins contributes to the hyperproliferation of glioma, and DNA replication is an excellent target to increase the sensitivity of glioma to radiotherapy (Lim et al. 2020). And neovascularization is a potent process for the development of malignancy, which owes to the VEGF signaling pathway. As its contribution to the growth and invasion of tumors (Zhao and Adjei 2015), VEGF/VEGFR pathway is brought to inhibition treatment, leading to rapid and long-lasting anti-angiogenesis and anti-tumor responses (Carmeliet and Jain 2011). And because VEGF is also a key factor in regulating glioma angiogenesis, the results of anti-VEGF therapy showed improved progression-free survival of relapsed GBM (Robles Irizarry et al. 2012). Enrichment in focal adhesion and Toll-like receptor signaling pathway also remind us that *DRAXIN* may partake in migration and tumor immunity of glioma. From the above conclusions, it can be inferred that the mechanism of *DRAXIN* in the poor prognosis of glioma depends on synergy of multiple pathways.

As itself, genes with similar functions or variations are somewhat linked and will have a tendency to co-express, which can be used to screen genes with similar sequences (Cheng et al. 2008). Hence, by co-expression analysis of the data in the CGGA-seq database, the ten most positively and negatively co-expressed genes of *DRAXIN* were shown separately, among which, *CDCA8* gene is the one with the strongest positive association to *DRAXIN*. According to previous studies, *KIF23*, a member of the kinesin family positively associated with *CDCA8*, is involved in the regulation of cytoplasmic division, and its high expression is associated with poor prognosis of gliomas (Gao et al. 2020). The idea mentioned above may give us an inspiration to *DRAXIN* standing in glioma. On the other hand, *ETNPPL*, a gene inversely associated with *DRAXIN*, is also negatively associated with the grade of glioma as its expression is not detected in glioblastomas. Overexpression of *ETNPPL* protein can exhibit tumor suppressive effect and inhibit the proliferation of glioma stem cells (Leventoux et al. 2020). Other co-expressed genes are also involved in the progression of glioma, such as *KIF2C* (Bie et al. 2012), *DLGAP5* (Zhou et al. 2021) and *CCNB1* (Yang et al. 2020)*.* The above biomarkers show a coordinated association with glioma progression, indicating that their co-expressed gene *DRAXIN* is inevitably functional in the development of glioma.

This study not only contains the predicted results from the above bioinformatics, but also validates the exact function of *DRAXIN* in glioma using in vitro experiments. As one of the most fundamental features of gliomas, abnormal proliferation can lead to continuous tumor progression, crowding out the viability of normal brain tissue and leading to immature differentiation of neoplastic cells, further aggravating the malignancy of gliomas (Caglayan et al. 2013). It has been reported that expression of S100A11, which correlates with glioma pathological grade, promotes the proliferation and tumorigenic capacity of glioma cells, and knockdown of S100A11 expression using shRNA results in the opposite phenotype (Wang et al. 2021). Similarly, knockdown of *DRAXIN* was demonstrated in this experiment to significantly inhibit the proliferative capacity of U251 cells. Furthermore, the aggressiveness of glioma is a key indicator of its malignancy. Invasion into normal tissues will lead to unclear margins of glioma, making it difficult to remove surgically and laying the hidden danger of tumor recurrence (Tong et al. 2017). The present study also confirmed that *DRAXIN* could promote the invasion of glioma cells, as other oncogenes have been reported (Zhang and Ma 2021). The above conclusive evidence, combined with the results of the bioinformatics analysis, reveals the role of *DRAXIN* as a key pathogenic factor in glioma from multiple dimensions, suggesting that *DRAXIN* can be targeted for intervention to improve the prognosis of glioma patients.

In summary, we have studied a large amount of high-throughput sequencing data based on several databases, which reveals the association between *DRAXIN* and the prognosis of glioma for the first time. However, there are some shortcomings in this study worth noting. Foremost, due to different medical environments and data processing methods, clinical information is collected based on different standards, resulting in unsatisfactory consistency of the data profiles. However, we collected data from several public databases with a large amount of glioma samples, to the best elimination of data bias. Secondly, the statistical results may be affected by the lack of normal samples in the database, which has a large gap with the tumor sample size. To compensate for these shortcomings, we used RT-qPCR and IHC to verify the differences in *DRAXIN* expression at the tissue level and cellular level. And numerous analysis methods also make a guarantee for accurate results.

## Conclusion

Collectively, for the first time, *DRAXIN* was found to be highly expressed in gliomas as an oncogene leading to shorter survival and poor prognosis of patients. In addition, *DRAXIN* may be involved in the cell cycle and VEGF pathway to affect the progression of gliomas. Moreover, the role of *DRAXIN* in promoting glioma proliferation and invasion has also been demonstrated. This study provides a molecular basis for subsequent studies on *DRAXIN* as a potential biomarker to predict prognosis of glioma patients and also a therapeutic target for glioma therapy.

## Data Availability

Please contact the authors for data requests.

## Abbreviations

RT-qPCR: Reverse transcription polymerase chain reaction
GEO: Gene Expression Omnibus
GEPIA: Gene Expression Profiling Interactive Analysis
*Draxin*: Dorsal Inhibitory Axon Guidance Protein
CGGA: Chinese Glioma Genome Atlas
GSEA: Gene Set Enrichment Analysis
ROC: Receiver operating characteristic
OS: Overall survival
IDH: Isocitrate dehydrogenase
GBM: Glioblastoma
VEGF: Vascular endothelial growth factor
CCNU: Chloroethylcyclohexylnitrosourea
